# Proximate, Mineral and Antinutrient Contents of Cocoyam (*Xanthosoma sagittifolium *(L.) Schott) from Ethiopia

**DOI:** 10.1155/2019/8965476

**Published:** 2019-11-19

**Authors:** Eyasu Wada, Tileye Feyissa, Kassahun Tesfaye

**Affiliations:** ^1^Department of Biology, College of Natural Sciences, Wolaita Sodo University, P.O. Box 138, Wolaita Sodo, Ethiopia; ^2^Institute of Biotechnology, College of Natural Sciences, Addis Ababa University, P.O. Box 1176, Addis Ababa, Ethiopia

## Abstract

Cocoyam (*Xanthosoma sagittifolium* (L.) Schott) is an important food crop especially in the tropics and subtropics. Its cormels and leaves are eaten after cooking in the rural areas in Ethiopia. There is lack of information on the nutritional composition of cocoyam grown in the country. In this study, cormels of green- and purple- cocoyams were analyzed to determine proximate and mineral contents and antinutritional factors. The moisture contents (%) of green- and purple-cocoyams were 61.91 and 63.53, respectively. Crude protein (10.10%) and fiber (2.66%) contents of purple cocoyam were significantly higher than crude protein (8.48%) and fiber (2.14%) contents of green cocoyam. Fat contents (%) of the green- and purple cocoyam were 0.85 and 0.22, respectively. Ash content of green cocoyam (3.25%) was significantly higher than the ash content of purple cocoyam (2.27%). The carbohydrate contents (%) and gross energy values (kcal/100 g) of green- and purple-cocoyam, respectively, were 85.36 and 378.47 and 84.76 and 380.27, showing that cocoyam grown in Ethiopia can be a good source of energy. Mineral contents (mg/100 g) of green cocoyam were determined as Fe (8.20), Zn (3.07), Cu (1.04), Mg (78.77), Mn (2.48), P (120.93), Na (29.22), K (1085.70) and Ca (56.57) while purple cocoyam had Fe (9.88), Zn (3.12), Cu (1.14), Mg (82.00), Mn (3.74), P (129.87), Na (24.33), K (1223.30) and Ca (44.90). High antinutritional factors (phytate and tannin) (mg/100 g) were determined from both green- and purple-cocoyam genotypes with significantly higher quantities in purple cocoyam (187.57 phytate and 156.1 tannin) than the green cocoyam (167.76 phytate and 139.62 tannin). This study provided important information about the nutritional composition of cocoyam from Ethiopia, which can help to develop cocoyam food products and to promote production and utilization of cocoyam by encouraging its sustainable use. More detailed analyses including processing and sensory testing are suggested for further investigation in order to obtain healthful and comfortable cocoyam products.

## 1. Introduction

Humankind has used over 7000 edible plant species, at one time or another. Research, however, has concentrated on a few crops to meet the food needs. Over 50% of humankind's requirements for calories and protein are met by just three crops (maize, wheat and rice) and 95% of the world's food energy needs are provided by just about 35 crop plant species [1]. Many plant species with a considerable importance for food security are categorized under neglected and underutilized crops. Some researchers have provided data to confirm the nutritional superiority of neglected and underutilized crops and their wild varieties over other more extensively utilized crops. Root and tuber crops are staple foods in many countries and are considered a good and inexpensive source of energy in the diets [2]. They are often produced with very low inputs but contribute greatly to food security and are culturally held in high esteem [3].

Aroids are grouped with the neglected and underutilized crops which over the years have received little research attention [4] although they are important tuberous root crops playing a significant role in the livelihood of millions of relatively poor people in developing countries [5]. The most important food aroids are from tribes *Colocasieae *and *Caladieae*,i.e., taro (*Colocasia esculenta *(L.) Schott)and cocoyam (*Xanthosoma sagittifolium *(L.) Schott). They are often considered jointly and many developing countries depend on these aroids as a source of carbohydrates and they are important food for more than 400 million people around the world [6].

Cocoyam is reported to have superior nutritional value over major root and tuber crops, especially in terms of their protein digestibility and mineral composition [7]. In many tropical areas, cocoyam plays major role in the lives of many as a food security crop, mainly for smallholder farmers. Cocoyam has overtaken taro (related aroid), in terms of proximate and mineral contents [8, 9]. Cormels of cocoyam are boiled, baked or partly boiled and fried in oil before consumption. The corms are peeled, dried and ground to flour for pastry that can be stuffed with meat or other fillings [10]. The young leaves can be boiled and used as vegetable similar to spinach [11].

In Ethiopia, cocoyam is expanding to new areas, growing even in poor soils and under dry conditions [12, 13]. The cultivation of cocoyam is increasing in the country. The cormels are eaten by cooking using pots or roasting using stones. The young leaves of green cocoyam are edible in some areas of southwestern parts of the country. Studies on varieties of cassava, sweet potato and yam showed that there are great differences in nutrient content within species, and that some varieties can provide a substantial contribution to nutritional requirements, not only for energy but also for protein and micronutrients [2]. In Ethiopia, both green- and purple-colored cocoyam genotypes are mainly used for food and fodder/feed while the purple-colored cocoyam is also used as medicine to treat Wulawushiya (related with hepatitis),Barqa (postpartum depression) and Gergeda (related with rheumatoid arthritis) [14]. The data on the nutritional composition of cocoyam is much less than that for other root and tuber crops and there is a paucity of data on the nutritional compositions of cocoyam growing in the country. There is a need to analyze, compile and disseminate data on the nutritional composition of Ethiopian cocoyam. The main aim of this study was, therefore, to determine proximate composition (moisture, crude protein, crude fiber, crude fat, total ash, total carbohydrate and gross energy), minerals such as Iron (Fe), Zinc (Zn), Copper (Cu), Magnesium (Mg), Manganese (Mn), Phosphorus (P), Sodium (Na), Potassium (K) and Calcium (Ca), and antinutritional factors (phytate and tannin) of green- and purple-cocoyam grown in Ethiopia and to compare the difference.

## 2. Materials and Methods

### 2.1. Sample Collection

Fresh cormels (small, middle and large sizes) that were not attacked by pests and which were not damaged during harvesting were selected from green-and purple-cocoyam, after 9 months of plantation.

### 2.2. Preparation of Cocoyam Flour

Cormels of three size groups (small, medium and large) were carefully selected from green- and purple-cocoyam (one accession from each) for purpose of including the size groups. The selected samples were washed using running tap water. Then hand peeled using stainless steel knife, washed and sliced to uniform thickness (~5 mm). The slices were blanched in hot water (80°C) for 5 min followed by immediate cooling in cold water in order to inactivate enzymes that may cause browning. The slices were placed on a stainless-steel tray and dried overnight in a dry oven at 105°C. The dried cormels chips were grinded using mortar and pestle to convert into flour. Then the flour was filled in polyethylene bags, packed and kept in desiccators until analyzed for contents of proximate, mineral and antinutritional factors.

### 2.3. Determination of Proximate Composition

Proximate composition (total moisture content, crude protein, crude fat, crude fiber, total ash, total carbohydrate and gross energy values) of the two types of cocoyams were determined by the following methods.

#### 2.3.1. Determination of Moisture Content

Moisture content (%) was determined in an oven drying methods at 105 ± 5°C according to the procedure described in Association of Official Analytical Chemists [15]. Five grams of each fresh sample was accurately weighed in triplicate and placed in a pre-weighed aluminum dish and dried in an oven at 105 ± 5°C till the constant weight of dry matter was obtained. The moisture content in the sample was determined as:

(1) $$\begin{array}{c} \text{Moisture}\;\left(\%\right)=\frac{\text{Wt}.\;\text{of}\;\text{fresh}\;\text{sample}-\text{Wt}.\;\text{of}\;\text{dried}\;\text{sample}}{\text{Wt}.\;\text{of}\;\text{fresh}\;\text{sample}}*100. \end{array}$$

#### 2.3.2. Determination of Crude Protein

The powdered cormel samples were analyzed for crude protein content according to the Kjeldahl's method described in the Association of Official Analytical Chemists [15].

*Protein Digestion*. Five grams of the sample was weighed in an ash less filter paper and put into 250 ml digestion flask. Then 3 g of a catalytic mixture, tablet (75 g of CuSO_4_ and 0.7 g of K_2_SO_4_) and 15 mL of 98% H_2_SO_4_ were added into a digestion flask. The whole mixture was subjected to heating in a digestion chamber until transparent residue (clear light green) content was obtained. Then, it was allowed to cool. After cooling, the digest was transferred into a 100 mL volumetric flask and made up to the mark (100 mL) with distilled water and then distilled using distillation apparatus.

*Protein Distillation*. Before use, the distillation apparatus was steamed for 15 min. After which, 100 ml conical flask containing 20 ml of 40% boric acid and 2 or 3 drops of Tashiro's indicator was placed under the distillation apparatus with its out let tubes inserted into the conical flask. The digest was washed down with distilled water followed by addition of 3–4 drops of phenolphthalein and 20 mL of 40% (w/v) NaOH solution. The distillation was continued until about 25 mL of distillate was trapped into the boric acid plus indicator solution changed from red to light grey, showing that all the ammonia liberated had been trapped. That means the digest in the condenser was steamed through until enough ammonia gas captured by the boric acid.

*Titration*. The solution in the receiving flask was titrated with 0.1 mM HCl to a brown color. After titration the % of nitrogen was calculated as:

(2) $$\begin{array}{c} \text{Nitrogen}\;\left(\%\right)=\frac{\left(\text{Vs}-\text{VB}\right)*\text{mM}\;\text{HCl}*0.014008}{\text{Wt}.\;\text{of}\;\text{sample}}*100, \end{array}$$

where Vs = Volume (mL) of HCl required to titrate sample; VB = Volume (mL) of acid required to titrate the blank; mM acid = Molarity of acid; *W* = Weight of sample (g). Then, percentage of crude protein in the sample was calculated from the % nitrogen as:

(3) $$\begin{array}{c} \text{Crude}\;\text{protein}\;\left(\%\right)=\;\%\;\text{N}\times F, \end{array}$$

where, *F* (conversion factor) is equivalent to 6.25 [15]. A blank was run through along with the sample and triplicate analysis was conducted for samples.

#### 2.3.3. Determination of Crude Fiber

Six gram of powdered sample (*E*) was taken into 50 mL tube and 2.5 mL of alpha-amylase was added and incubated at room temperature for 10 min. Then, 60 mL of a mixture composed of 700 mL 70% acetic acid, 100 mL 65% nitric acid and 20 g trichloroacetic acid was added. Digestion was undertaken in 250 mL flask by heating at 200°C by continuous string at 500 rpm for 30 min. Then after cooling on ice, filtrated with vacuum filtration on dry filter paper with known mass (*Mf*) by using distilled water until the filtrate became neutral. The residue on the filter paper was washed with 10 mL ethanol for 3 times and 10 mL acetone for 2 times to dissolve organic constituent. Then after transferring the dried residue with the filter paper into pre-weighted crucible, the residue was oven dried at 105°C overnight to drive off moisture. The oven dried crucible containing the residue and filter paper was cooled in a desiccator and weighted (*M*1). The residue and filter paper were burned first in Bunsen burner and then 550°C. The crucible containing white and grey ash (free of carbonaceous material) was cooled in a desiccator and weighted to obtain *M*2. The % of crude fiber was calculated as:

(4) $$\begin{array}{c} \text{Crude}\;\text{fiber}\;\left(\%\right)=\frac{\left(M1-Mf\right)-M2}{E}*100. \end{array}$$

#### 2.3.4. Determination of Crude Fat

The crude fat in the powdered samples was determined by automated Soxhlet extraction method [15]. After weighting the dried flask containing sand to constant weight, 15 g of homogenized samples were measured by using filter paper of known mass and placed in extraction flask. The dried flasks (250 mL) were weighed correspondingly and filled with 150 mL of petroleum ether. The extraction thimbles were plugged tightly with cotton wool and run for 2 h. The extraction chamber continuously filled with the sample there by extracting the fat. When the optimum sensor reached, the magnetic valve was opened and the samples were washed with freshly filled solvent (petroleum ether). Finally, the solvent was recovered by collection in solvent tank. The fat was collected in filter paper and the extract was gently evaporated to dryness. The remaining petroleum ether was removed by sonication. The extraction flask containing crude fat in the filter paper was dried in 105°C to constant weight. The % fat in the sample was calculated using the formula:

(5) $$\begin{array}{c} \text{Fat}\;\left(\%\right)=\frac{\text{Wt}.\;\text{of}\;\text{flask}\;\text{containing}\;\text{the}\;\text{crude}\;\text{fat}\;\text{in}\;\text{filter}\;\text{paper}-\text{Wt}.\;\text{of}\;\text{flask}\;\text{plus}\;\text{filter}\;\text{paper}}{\text{Wt}.\;\text{of}\;\text{sample}\;}*100. \end{array}$$

#### 2.3.5. Determination of Total Ash Content

A crucible was dried at 550°C for 30 min and cooled down in a desiccator for 1 h. The weight of crucible was measured (*M*1). Five grams of powdered sample was added in the dried crucible and the crucible containing sample was measured (*M*2). Then the sample was burned by using Bunsen burner until the steam off and then in oven at 550°C for 5 h. Ash is an inorganic residue remaining after the material has been completely burnt. The crucible containing ash was cooled in a desiccator and then re-weighted (*M*3) [15]. The % of ash contents in the cocoyam sample was calculated as:

(6) $$\begin{array}{c} \text{Ash}\;\left(\%\right)=\frac{M3-M1}{M2-M1}*100. \end{array}$$

#### 2.3.6. Determination of Total Carbohydrate

Total carbohydrate content was calculated adding the total values of crude protein, crude fat, crude fiber and total ash contents of the sample and subtracting it from 100%.

(7) $$\begin{array}{c} \text{Total}\;\text{carbohydrate}\;\left(\%\right)\;=\;100\;-\;\left(\%\;\text{Crude fiber}\;+\;\%\;\text{Crude protein}\;+\;\%\;\text{Crude fat}\;+\;\%\;\text{Ash}\right). \end{array}$$

#### 2.3.7. Determination of Energy Value

Gross energy value (kcal/100 g) of the samples was determined by multiplying the protein content by 4, carbohydrate content by 4 and fat content by 9 [15]. 

(8) $$\begin{array}{c} \text{Energy}\;\text{value}\;=\;\left(\text{Crude}\;\text{protein}\;\times \;4\right)\;+\;\left(\text{Total}\;\text{carbohydrate}\;\times \;4\right)\;+\;\left(\text{Crude}\;\text{fat}\;\times \;9\right). \end{array}$$

### 2.4. Determination of Mineral Content

Iron, Zinc, Copper, Magnesium, Manganese, Sodium and Potassium and Calcium were determined according to the standard method of AOAC [15] using an Atomic Absorption Spectrophotometer (Varian SAA-20 Plus). Ashing of the samples was followed by digestion and absorption. Phosphorus was determined by AAS method of AOAC [16].

### 2.5. Analysis of Antinutritional Factors

#### 2.5.1. Determination of Phytate

The phytate contents of green- and purple-cocoyam were determined according to method described by Latta and Eskin [17]. Dried sample of cocoyam flour (0.1 g) was extracted with 10 mL 2.4% HCl for 1 h at room temperature and centrifuged at 3000 rpm for 30 min. The clear supernatant was used for the phytate estimation. One ml of Wade reagent (0.03% solution of FeC1_3_·6H_2_O containing 0.3% sulfosalicylic acid in water) was added to 3 mL of the sample solution and the mixture was centrifuged. The absorbance at 500 nm was measured using UV–VIS spectrophotometer. The phytate concentration was calculated from the difference between the absorbance of the control (3 mL of water + 1 mL Wade reagent) and that of assayed sample and expressed as mg/100 g.

#### 2.5.2. Determination of Tannin

Tannin contents of green- and purple-cocoyam were determined using the method of Burns [18]. Cocoyam flour (0.25 g) was weighed in a screw capped test tube and 10 mL of 1% HCl in methanol was added to each test tube containing the samples. Then the tubes were put on mechanical shaker for 24 h at room temperature. After 24 h of shaking, the tubes were centrifuged at 1000 rpm for 5 min. One milliliter of the clear supernatant was taken and mixed with 5 mL of vanillin–HCl reagent in another test tube and this mixture was allowed to stand for 20 min to complete the reaction. After 20 min, the absorbance was read at 500 nm using spectrophotometer. The tannin concentration was calculated from the difference between the absorbance of control and that of the sample and expressed as mg/100 g.

### 2.6. Statistical Analysis

A comparative statistics and comparative analysis was conducted to present the difference in proximate composition (moisture, crude protein, crude fiber, crude fat, total ash, total carbohydrate and gross energy values), mineral contents (Ca, K, Na, Mg, Mn, Cu, Fe Zn and P) and antinutritional factors (phytate and tannin) in green- and purple-cocoyam samples. The analyses were performed using SPSS version 23 [19]. Differences in means at *p* < 0.05 were considered significant.

## 3. Results

### 3.1. Proximate Composition

The results of proximate analysis showed that the crude protein (10.10%), crude fiber (2.66%) and gross energy value (380.27 kcal/100 g) of purple cocoyam were significantly higher than the crude protein (8.48%), crude fiber (2.14%) contents and gross energy value (378.47 kcal/100 g) of the green cocoyam whereas the green cocoyam had showed significantly higher total ash content (3.25%) than the total ash content (2.27%) of purple cocoyam. Moisture (61.19%), fat (0.85%) and total carbohydrate (85.36%) contents of green cocoyam did not differ (*p* > 0.05) from the moisture (63.53%), total carbohydrate (84.76%) and fat (0.22%) contents of purple cocoyam (Table 1).

### 3.2. Mineral Composition and Antinutritional Factors

Nine different minerals and two antinutritional factors were analyzed for their concentration in dry weight basis (mg/100 g). The green cocoyam had Fe (8.20), Zn (3.07), Cu (1.04), Mg (78.77), Mn (2.48), P (120.93), Na (29.22), K (1085.70) and Ca (56.57) while purple cocoyam had Fe (9.88), Zn (3.12), Cu (1.14), Mg (82.00), Mn (3.74), P (129.87), Na (24.33), K (1223.30) and Ca (44.90). The result of the comparative analysis showed that Mg, Mn, P, Na, K and Ca contents of green cocoyam were significantly higher than Mg, Mn, P, Na, K and Ca contents of purple cocoyam. The Fe, Zn and Cu contents of green cocoyam were not significantly different from the Fe, Zn and Cu contents of the purple cocoyam. Significant quantities of antinutritional factors namely: phytate and tannin were found in both cocoyam morphotypes. High antnutritional factors were determined, where the phytate (187.57) and tannin (156.11) contents (mg/100 g) of purple cocoyam were significantly higher (*P* < 0.001) than the phytate (167.76) and tannin (139.62) contents of green cocoyam (Table 2).

## 4. Discussion

This study provided information about proximate and mineral and antinutrient (phytate and tannin) contents of neglected and underutilized crop species, green- and purple-cocoyam, from Ethiopia. The moisture content of the green cocoyam was 61.91% and that of the purple cocoyam was 63.53%. These values were within the range of moisture contents of cocoyam from Ghana [20]. The moisture contents of cocoyam reported from Tanzania and Uganda [9] and Nigeria [21] were 68.41% and 68.28%, respectively. The relative low moisture content was determined from cocoyam in this study in comparison with other studies [22] can be helpful for the storage of cocoyam cormels at ambient temperature.

The crude protein content of green cocoyam (8.48%) and purple cocoyam (10.10%) determined by this study were higher than mean protein contents (4.75%) of cocoyam reported from Tanzania and Uganda [9] and Nigeria [23]. The crude protein contents were also higher than the crude protein contents of from the Assam State of India (2.42%) [5]. Lu et al. (2005) [24] reported that the protein content of isolated starches ranged from 0.04% to 0.06%. Higher mean protein contents of cocoyam determined in the study may be due to cormel samples (not corms) were used for protein determination. The results were comparable with cocoyam cormles protein contents of Ede Uhie (8.08%) and Ede Ocha (8.74%) cocoyam varieties from Nigeria [25]. The present study results were also in agreement with a review conducted by Shewry [26] who reported that plant tubers contain protein up to 10%. According to Akpan and Umon [27], the protein content of cocoyam was slightly superior to taro. This study results showed that both green- and purple- cocoyams are rich in proteins, which can be considered as good opportunity since it is mainly consumed by resource poor farmers.

The crude fiber contents of green cocoyam (2.14%) and purple cocoyam (2.66%) determined in this study were lower than the minimum crude fiber content (2.80%) of two related aroids (*Colocasia *and *Xanthosoma*) from Assam State of India [5]. The crude fiber contents were, however, higher than the range of crude fiber contents of cocoyam (0.6–1.9%) reported by Akpan and Umoh [27] and within the range of the crude fiber content (1.11–3.00%) of cocoyam reported by Sefa-Dedeh and Kofi-Agyir [20]. The purple cocoyam had significantly higher crude fiber content (2.66%) than the crude fiber content (2.14%) of green cocoyam (*p* < 0.01). In contrast to this, Sarma et al. [5] reported the crude fiber content of purple cocoyam (2.80%) was lower than the crude fiber content (3.50%) of dark green leaved cocoyam. The present study result showed that fiber contents in green- and purple-cocoyam grown in Ethiopia can be effective and useful source of fiber. The differences in the fibers contents could be attributed to the genotype difference.

Comparative analysis showed that the crude fat content of green cocoyam (0.85%) was not significantly different from the fat content of purple cocoyam (0.22%) (*p* > 0.05). The values were relatively comparable with the average crude fat contents of cocoyam reported by Akpan and Umoh [27] (0.93%) and Ndabikunze et al. [9] (0.43%), indicating cocoyam is a low-fat crop. Thus, it can be said to be preferred food crop that can contribute less to the health problems related with excess fat intake.

The total ash content of green cocoyam (3.25%) was significantly higher than the total ash content of purple cocoyam (2.27%) (*p* < 0.001). The values were higher than the total ash content of mean ash content of cocoyam from southern Nigeria ranged from 0.88% to 1.15% [28]. The values were comparable with the total ash content of Ede Ofe (3.00%) and Ede Ocha (2.45%) cocoyam cultivars from Nigeria [25], but lower than the total ash content of white-fleshed (5%), yellow-fleshed (4.6%) and red-fleshed (4.5%) cocoyam varieties reported latter by Ihediohanma et al. [29] from the same country. The values were also smaller than the total ash contents of cocoyam (3.51%) grown along the Lake Victoria Basin in Tanzania and Uganda [9]. The differences could be the influence of the environments in which cocoyams were grown.

The total carbohydrate contents of green cocoyam (85.36%) and purple cocoyam (84.76%) were comparable with total carbohydrate content (85.65%) of released taro variety from Ethiopia (Boloso I) [30]. Gross energy values (kcal/100 g) of green cocoyam (378.47) and purple cocoyam (380.27) were relatively higher than the gross energy values (kcal/100 g) of Boloso I, which varied from 370 to 374 kcal/100 g [30]. The carbohydrate contents and gross energy values indicate that cocoyam growing in Ethiopia is one of carbohydrate rich foods in supplying high energy.

The minerals such as Fe, Zn, Cu, Mn, Na and K contents (mg/100 g) of green- and purple- cocoyams were higher than the Fe, Zn, Cu, Mn, Na and K content of cocoyam grown along the Lake Victoria Basin in Tanzania and Uganda [9]. The Mg, P and Ca (mg/100 g) contents of cocoyam were lower than Mg (90.62), P (207.50) and Ca (110.17) contents of cocoyam reported by Ndabikunze et al. [10]. The Cu content of green cocoyam (1.04) and purple cocoyam (1.14) were higher than the Cu contents of white-fleshed cocoyam (0.52), red-fleshed cocoyam (0.78) and taro (0.78) cultivars reported by Njoku and Ohia [31]. The present study showed that both green- and purple- cocoyams are rich in minerals. According to Njoku and Ohia [31], consumption of nutrient rich foods such as cocoyamhelps the body to utilize protein, carbohydrates and other nutrients.

High phytate concentrations (mg/100 g) observed in green cocoyam (167.76) and purple cocoyam (187.57). Phytate has been recognized as an anti-nutrient due to its adverse effects because it lowers the availability of many minerals such as copper, iron and zinc [32]. The tannin levels (mg/100 g) of green- and purple- cocoyams were 139.62 and 156.11, respectively. These tannin contents were higher than the tannin content of taro-Boloso I reported by Adane Tilahun et al. [30]. It is known to exert negative effect on the bioavailability of proteins and minerals [30]. Various studies have shown that the processing methods such as boiling, fermentation and roasting can significantly reduce antinutritional factors (phytate and tannin) to low level [30, 33, 34]. These processing methods have to be tested for the reduction of antinutritional contents of cocoyam grown in Ethiopia.

## 5. Conclusion

This study addressed the knowledge gap that existed in cocoyam, particularly the paucity of scientific data, providing information on proximate and mineral contents and antinutritional factors of green- and purple-colored cocoyams growing in Ethiopia. The analysis showed that both morphotypes of cocoyam can provide nutrient-rich products, with slight differences in the quantities of proximate and minerals contents. The cocoyam would play a significant role in alleviating the household food insecurity and periodic food shortages existing in some families inhabiting cocoyam growing areas although the high antinutritional factors would affect its acceptance. More detailed analyses including processing and sensory testing, protein quality analysis and determining phytochemical and antioxidants from extracts are suggested for further investigation in order to obtain healthful and comfortable cocoyam products.

## Figures and Tables

**Table 1:** Proximate composition of green- and purple-cocoyams.

Proximate	Green cocoyam	Purple cocoyam	Significance
Moisture (%)	61.91 ± 2.50	63.53 ± 1.10	0.372
Crude protein (%)	8.48 ± 0.36	10.10 ± 0.18	^∗^
Crude fiber (%)	2.14 ± 0.12	2.66 ± 0.09	^∗∗^
Crude fat (%)	0.85 ± 0.60	0.22 ± 0.10	0.211
Total ash (%)	3.25 ± 0.09	2.27 ± 0.07	^∗∗^
Total carbohydrate (%)	85.36 ± 0.49	84.76 ± 0.38	0.195
Energy value (kcal/100 g)	378.47 ± 0.71	380.27 ± 0.43	^∗^

Data presented are independent sample *t*-test. Values are means of triplicates analysis ± standard deviations. Value in level of significance are ^∗∗^*p* < 0.01, ^∗^*P* < 0.05 and mean values with *p* > 0.05 are not significantly different and their respective *p*-values are shown.

**Table 2:** Mineral contents and antinutritional factors of green- and purple-cocoyams.

Minerals (mg/100 g)	Green cocoyam	Purple cocoyam	Significance
Fe	8.20 ± 0.6	9.88 ± 1.97	0.305
Zn	3.07 ± 0.10	3.12 ± 0.12	0.582
Cu	1.04 ± 0.08	1.14 ± 0.05	0.183
Mg	78.77 ± 0.67	82.00 ± 1.04	^∗^
Mn	2.48 ± 0.19	3.74 ± 0.06	^∗∗^
P	120.93 ± 2.07	129.87 ± 2.06	^∗^
Na	29.22+1.44	24.33 ± 0.82	^∗^
K	1085.70 ± 32.1	1223.30 ± 28.70	^∗^
Ca	56.57 ± 1.50	44.90 ± 1.81	^∗∗^

*Antinutritional factor*			
Phytate	167.76 ± 2.82	187.57 ± 0.55	^∗∗∗^
Tannin	139.62 ± 0.97	156.11 ± 2.35	^∗∗∗^

Data presented are independent sample *t*-test. Values are means of triplicate analysis ± standard deviations. Value in level of significance are ^∗∗∗^*p* < 0.001, ^∗∗^*p* < 0.01, ^∗^*p* < 0.05 and mean values with *p* > 0.05 are not significantly different and their respective *p*-values are shown.

## Data Availability

The proximate, minerals, and antinitritional factors' data used to support the findings of this study are included within the article.
